# BK virus associated meningoencephalitis in an AIDS patient treated with HAART

**DOI:** 10.1186/1742-6405-4-13

**Published:** 2007-06-08

**Authors:** José E Vidal, Maria C Fink, Filiberto Cedeno-Laurent, Serena Delbue, Pasquale Ferrante, Rafi F Dauar, Francisco Bonasser Filho, Roberta Schiavon Nogueira, Eduardo E Calore, Claudio S Pannuti, J Roberto Trujillo, Augusto C Penalva de Oliveira

**Affiliations:** 1 Department of Infectious Diseases, Emilio Ribas Institute of Infectious Diseases, São Paulo, Brazil; 2 Department of Neurology, Emilio Ribas Institute of Infectious Diseases, São Paulo, Brazil; 3 Department of Neurosurgery, Emilio Ribas Institute of Infectious Diseases, São Paulo, Brazil; 4 Department of Pathology, Emilio Ribas Institute of Infectious Diseases, São Paulo, Brazil; 5 Department of Infectious Diseases, São Paulo University, São Paulo, Brazil; 6 Laboratory of Virology of the Institute of Tropical Medicine, São Paulo University, Brazil; 7 Laboratory of Molecular Medicine and Biotechnology of Don C. Gnocchi Foundation ONLUS, IRCCS, Milan, Italy; 8 Department of Technology and Biomed Sciences University of Milan, Italy; 9 Clinical Research Unit on Human Retrovirology, University of Campinas, São Paulo, Brazil; 10 Institute of Human Virology, University of Maryland Biotechnology Institute, UMD, USA

## Abstract

A severely immune-suppressed AIDS patient was suspected of suffering from BK virus (BKV) meningoencephalitis, after being studied for common causes of neurological complications of co-infectious origin. Polymerase chain reaction (PCR) and sequence analysis of cerebrospinal fluid and brain samples, confirmed the presence of BKV. His clinical condition improved along with the regression of brain lesions, after modifications on his antiretroviral regime. Five months after discharge, the patient was readmitted because of frequent headaches, and a marked inflammatory reaction was evidenced by a new magnetic resonance imaging (MRI). The symptoms paralleled a rising CD4^+ ^lymphocyte count, and immune reconstitution syndrome was suspected. This is the first non-postmortem report of BKV meningoencephalitis in an AIDS patient, showing clinical and radiographic improvement solely under HAART.

## Background

Neurological complications associated with HIV-1/AIDS are being recognized with a high frequency that parallels the increased number of AIDS cases [1]. Since the introduction of HAART, morbidity and mortality secondary to primary and secondary neurological opportunistic diseases in HIV-1/AIDS patients have significantly decreased [2,3]. However, neurocognitive impairments continue to occur in high frequencies, even in countries, with a free and universal access program to HAART, such as Brazil [4].

Recently, atypical presentations of brain diseases, such as JC virus granule cell neuronopathy, and those related to the reconstitution of the immune system after initiation of HAART have been reported in a growing basis [5-7]. These unusual neurological pictures in AIDS patients represent new diagnostic and therapeutic challenges [7,8].

BKV meningoencephalitis is a rare polyomaviral infection with fatal outcome when associated with AIDS [9-11]. The clinical picture is devastating, resulting in death from multi-organ failure [12]. Here, we describe the clinical course of an AIDS patient with presumed BKV meningoencephalitis who showed substantial improvement after modification of his HAART regime.

## Case presentation

In April 2004, a 43-year-old HIV-1 positive heterosexual male was admitted to the hospital complaining of bilateral headache of moderate intensity accompanied by speech, gait and memory disturbances. HIV-1 infection was diagnosed in July 2003 after an episode of cryptococcal meningitis; with a documented CD4^+ ^lymphocyte count of 6 cells/mm^3^. His past medical history includes an episode of pancreatitis secondary to lopinavir/ritonavir (July, 2003), and an episode of tuberculous meningitis (October, 2003). On admission, antiretroviral medications included zidovudine, lamivudine, and efavirenz (antiretroviral scheme modified after pancreatitis in August, 2003). Other medications included: fluconazole, TMP-SMX, isoniazid, ofloxacin, ethambutol, and pyrazinamide (rifampin was discontinued and replaced by ethambutol and ofloxacin after a marked increased of hepatic enzymes in November, 2003)

Initial examination revealed mental confusion, dysarthria and ataxia. Baseline cranial CT scan showed two hypodense lesions with mass effect and no contrast enhancement in the left temporo-parietal and right occipito-parietal areas. His CD4^+ ^lymphocyte count was 37 cells/mm^3, ^with an undetectable viral load. Presumptive cerebral toxoplasmosis was diagnosed, and a treatment with sulphadiazine-pyrimetamine, folinic acid, and dexamethasone was started. After 14 days of therapy, the patient's neurological status and CT scan findings remained unchanged. Analysis of the cerebrospinal fluid (CSF) obtained on day 14 after admission showed 12 leukocytes/mm^3 ^(79% lymphocytes, 10% monocytes), glucose of 46 mg/dl and a protein level of 146 mg/dl. Herpes simplex encephalitis was suspected, and acyclovir replaced the medications for toxoplasmosis. After a week on this therapeutic regime, the patient's neurological status remained unaltered, and PCR analysis of CSF was performed, resulting negative for all human herpes viruses, JCV, and for *Toxoplasma gondii*. Nevertheless, PCR for polyomavirus BKV resulted positive.

**Figure 1:**
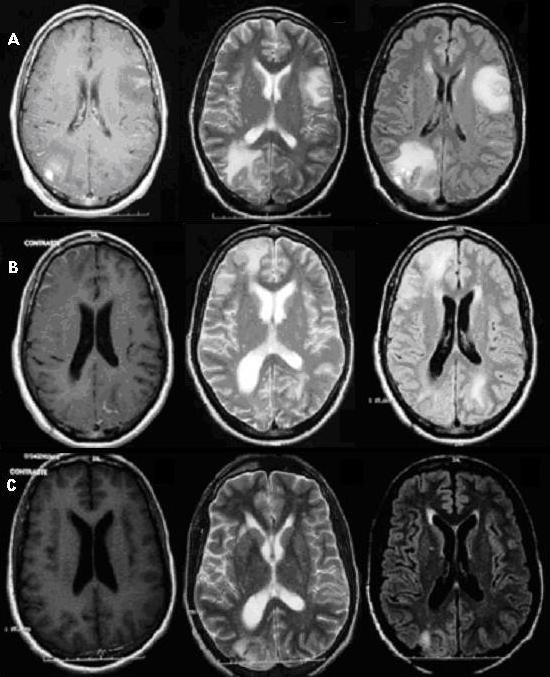
**Panel A**. Intraoperative brain images in April 2004. An axial T1-weighted image after gadolinium injection (left-hand side) shows lesions in the gray matter of the left temporo-parietal lobe and right occipital lobe (place of biopsy). These images show slight enhancement of the lesions particularly in the meninges and the presence of mass effect. An axial T2-weighted image (center) and a FLAIR image (right-hand side) show better details of the lesions. **Panel B**. Brain MRI images, 5 months after discharge. Axial T-1 weighted image after gadolinium injection (left-hand side) showed important improvement in gray matter lesions. However, images in T2-weighted (center), and FLAIR (right-hand side) showed presence of new high-signal-intensity lesions in the white matter of the right frontal lobe and left occipital lobe. A widening of the right ventricle compared to the figure in Panel A can be observed. **Panel C. **Brain MRI images, 7 months after discharge. Axial T-1 weighted image after gadolinium injection (left-hand side) shows normal appearance. Images in T2-weighted (center) and FLAIR (right-hand side) reveal regression of the white matter changes; however discrete widening of the right ventricle is still present.

**Figure 2:**
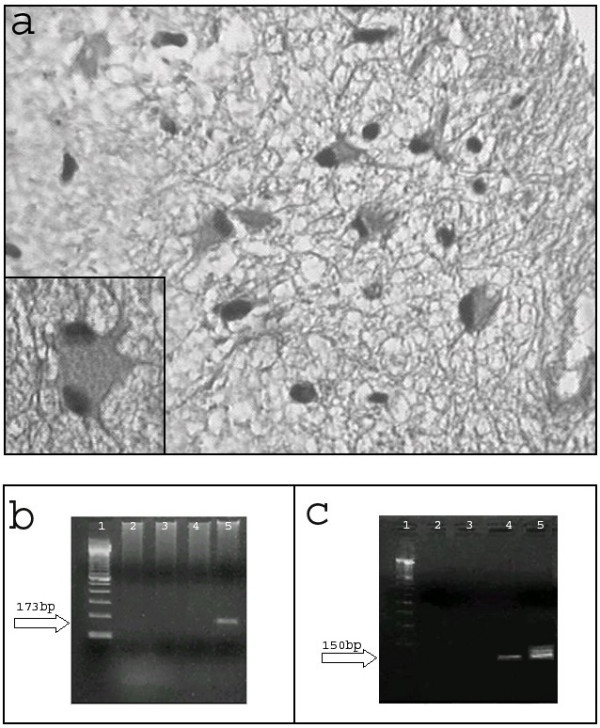
**A) **Representative section of the brain biopsy (H&E 40X) shows thickened leptomeninges with an infiltrate composed of lymphocytes and plasmocytes that extended perivascularly. Inset shows an astrocyte with prominent and hyperchromatic nuclei. **B) **Electrophoresis on 2% agarose gel of BKV PCR products from CSF. Lane 1, Marker 100 bp: Lane 2, negative control of PCR; Lane 3, negative control of extraction; Lane 4, 250 ng DNA; Lane 5, positive control of PCR. **C) **Electrophoresis on 2% agarose gel of JCV PCR products from brain biopsy. Lane 1, Marker 100 bp; Lane 2, negative control of PCR; Lane 3, negative control of extraction; Lane 4,1000 ng DNA; Lane 5, positive control of PCR.

From this data, acyclovir and dexamethasone were discontinued, and the patient underwent MRI-guided stereotactic brain biopsy of the lesion found in the right occipital lobe. Intraoperative MRI findings were similar to those previously seen by CT scan (Figure 1A). Histopathological examination showed thickened leptomeninges with a lymphocytic infiltrate that extended perivascularly (Figure 2A). The underlying cortex showed mild astrocytosis with prominent hypertrophic nuclei and bi-nucleated forms (Figure 2A). PCR examination of the brain tissue sample also showed the presence of BKV DNA in the absence of any other polyomaviruses (Figure 2C and 2D). The patient was reclassified as with presumptive BKV subacute meningoencephalitis. His therapeutic regime included the replacement of efavirenz for atazanavir-ritonavir along with the administration of zidovudine/lamivudine, leaving the patient exclusively with HAART. Urine analysis and renal function tests were performed, showing <10 leucocytes/field, undetectable red blood cells and proteins, a serum creatinine of 0.8 mg/dl, and a BUN of 16 mg/dl. These values, discarded the presence of renal and urinary abnormalities that are normally present in the context of BKV clinical infection. Four weeks after admission, neurological manifestations improved considerably, and the patient was discharged.

Five months after discharge, the patient complained of mild headaches. A new MRI reported improvement of the two lesions previously seen; however, new areas of increased signal intensity of the white matter appeared (Figure 1B). Based on a rising CD4^+ ^lymphocyte count of 144 cells/mm^3^, and in the absence of any other co-infectious agent, *"encephalopathy of unknown origin" *secondary to immune reconstitution was suspected. We kept close follow-up without any other intervention, and the patient gradually recovered.

Seven months after discharge, MRI lesions and CD4^+ ^lymphocyte count further improved (Figure 1C.). Since then, and until May 2007, the patient has been in the same HAART regime, with no neurological complaints; his renal and liver function have always been unaltered, and his last CD4 ^+ ^lymphocyte count was of 336 cells/mm^3 ^with a viral load bellow 400 copies/ml.

## Methods

### Serum Hiv-1 viral load detection

All quantifications of viral load were performed by real-time RT-PCR. The threshold of detection was 400 copies/ml.

### BKV and JCV DNA amplification from CSF samples

PCR technique as described by Arthur *et. al*.,[13] was used to amplify the T-antigen gene (173 bp) of both, BKV and JCV. To establish whether BK viral sequences inhibited the reaction, 10 μl of CSF sample were added to duplicate tubes containing the PCR mixture and polyomavirus cloned DNA. An amplification band of 173 bp was analyzed by 2% agarose gel electrophoresis and visualized by exposure to UV light after staining with ethidium bromide. Discriminating between JCV and BKV was performed by treatment of PCR products (10 μl) with the restriction enzyme BamH1 (Invitrogen) and analyzed by electrophoresis on a 3% agarose gel.

### BKV and JCV DNA amplification from brain biopsy

DNA was isolated twice from 25 mg of paraffin-embedded tissue using the DNeasy Tissue Kit (QIAGEN, USA). Negative control of extraction was carried out, using water instead of lysed tissue sample. DNA amplification was carried out by seminested-PCR on different amount of DNA purified from paraffin-embedded biopsies. A 150 base pair fragment of the BKV LT-coding region was amplified using the outer primers Pep1 and Pep2 and the inner primers Pep1 and BKS (nt 4513–4529, Dunlop strain). Parameters for the 30 cycles of outer PCR were 95°C denaturation, 54°C annealing and 72°C elongation for 30 sec each, whereas for the inner PCR the annealing temperature was 52°C. Urine from a patient with "polyomavirus associated nephropathy" was used as positive control, while water as negative control. Amplification of the LT region of JCV DNA was carried out by nested-PCR on different amount of DNA, by using set of primers JC1 and JC2 (outer), Pep1 and Pep 2 (inner). Moreover, to discard contamination by other polyomavirus, we performed a western blot analysis with the antibody against JCV/SV40 Tag (94 kDa) and against actin (40 kDa) as a control. Our patient's sample came out negative for the former (data not shown).

### Sequence analysis

PCR products were added to a mixture containing 4 μl of *Ready Reaction Premix *2.5X, 2 μl of *BigDye Sequencing Applied Biosystems *5X buffer, 3.2 pmol of either *forward or reverse primers*, and water up to a final volume of 20 μl. The cycle sequencing was performed using the GeneAmp PCR *System *9700, with the following protocol: initial denaturation at 96°C for 1', then 25 cycles with a first step at 96°C for 10", a second step at 50°C for 5" and the last rapid thermal ramp to 60°C for 4'. 5 μl of the purified product underwent electrophoresis on an ABI PRISM 310 Genetic Analyzer. Sequence homology searches were performed using BLAST at NCBI (USA).

## Conclusion

We report the first *in-vivo *case of BKV-associated meningoencephalitis in an AIDS patient who showed clinical improvement and regression of brain lesions while on HAART.

BKV infection is generally a benign condition in immunocompetent patients, and the causative agent of "polyomavirus associated nephropathy" and hemorrhagic cystitis in immunocompromised individuals [12,14]. However, there is a growing body of evidence demonstrating its neurotropism [14-16]. Among AIDS patients, only 3 cases of BKV- meningoencephalitis have been described, and all of them reported post-mortem [9-12].

Initially, the morphology of the lesions and the cytochemical profile of the CSF samples led us to empirically diagnose the patient with cerebral toxoplasmosis and herpes simplex encephalitis afterwards. However, the lack of response to treatment and the presence of BKV DNA in CSF samples and brain tissue led us to reclassify the patient as with BKV meningoencephalitis.

Compared to the other 3 cases reported, the presence of BKV DNA in the CSF is a common denominator of true neurological disease. As previously described, the detection of BKV genome in brain samples is unspecific, as it is normally present in 3%–6% of HIV-1 infected patient, even without neurological symptoms [17]. Our patient's histopathological description is identical to the ones previously reported, showing diffuse areas of increased signal [9,11]. Progressive multifocal leukoencephalopathy was ruled out because of the normal morphology of oligodendrocytes. In terms of the radiological changes, our case is similar to the previous ones, showing areas of increased signal intensity of the periventricular white matter (MRI) [11]. Other descriptions include increased meningeal contrast enhancement along with increased meningeal thickness (MRI) [9], and marked internal hydrocephalus and periventricular lucencies (CT scan) [10]. None of the previous reports showed features suggesting immune reconstitution inflammatory syndrome (IRIS).

Our main limitation to conclude that BKV is the causative agent of the neurological disorders is the lack of demonstration of the virus in the brain tissue sample, either by immunohistochemistry or by *in-situ *hybridization. Tissue samples extracted by stereotactic surgery are limited, and most of the times insufficient to run all diagnostic tests. These limitations were not faced in other reported cases, as all of these were done post-mortem.

Another important difference is the lack of renal/systemic involvement in our patient, when compared to the other cases previously reported. Our patient' renal work up included serial urianalysis and renal function tests that remained normal throughout the course of the disease (creatinine 0.8 mg/dl, BUN of 16 mg/dl, in April, 2004, creatinine of 0.9 mg/dl, BUN of 28 mg/dl in October, 2004,). As an outpatient his renal function has continued in the normal ranges (creatinine of 0.9 mg/dl, BUN of 40 mg/dl, May 2007). The containment of the disease was possibly related to a partial effect of HAART, similar to what has been reported for other AIDS-associated neurological complications in the post-HAART era, opening a broader spectrum of AIDS-associated neurocognitive disorders [18].

In this case, the change of atazanavir-ritonavir for efavirenz might have helped to reactivate the immune system, improving the symptoms and morphology of the lesions associated with BKV meningoencephalitis. The rationale of this change was based upon our previous experience and the results of an unpublished work describing the benefits of protease inhibitors in the restoration of the CD4 count when compared to non-nucleoside analogs *(Riddler SA, Haubrich R, DiRienzo G, et al. A prospective, randomized, phase III trial of NRTI-, PI-, and NNRTI-sparing regimens for initial treatment of HIV-1 infection: ACTG 5142. Program and abstracts of the XVI International AIDS Conference; August 13–18, 2006; Toronto, Canada. Abstract THLB0204*). Unfortunately, the restoration of the immune system is not innocuous, and as previously described, deleterious effects are found in the context of polyomavirus infection, introduction of HAART, and a rising CD4^+ ^count [19]. Immune reconstitution inflammatory syndrome (IRIS), as the latter phenomenon is known, affects 15% to 45% of patients receiving HAART. CNS involvement hás been reported to occur in the presence of tuberculosis (33%), cryptococcosis (4.2%–15.9%), JC vírus (unknown frequency); being manifested radiologically as the extension or worsening of a previous condition or by the apparison of lesions that are enhanced by contrast, with or without involvement of the meninges and sometimes accompanied by hydrocephalus [6]. In our patient, the newly developed cerebral lesions and the headaches spontaneously resolved.

In conclusion, our report demonstrates that BKV might be the causative agent of meningoencephalitis in AIDS patients, and that this virus must be investigated in higher frequencies in HIV-1 positive patients with neurological manifestations now that its neurotropism has been better documented. In our patient, a previously reported life-threatening and disseminated disease progressed to a mild and focalized neurological condition in the presence of HAART.

## Competing interests

The author(s) declare that they have no competing interests.

## Authors' contributions

JEV was in charge of the inpatient/outpatient care, management collected and analyzed all the data, drafted the manuscript, and made critical intellectual contributions; MCF performed the PCR for BKV and JCV in CSF, and BKV sequence in CSF, and drafted the manuscript; FCD participated in the design and drafting of manuscript, collected the data, and made critical intellectual contributions; SD performed the PCR for BKV and JCV in brain tissue, performed the western blot of cross-reactivity for SV40, JCV and BKV proteins, and drafted the manuscript; PF performed the PCR BKV and JCV in brain tissue, performed the western blot of cross-reactivity for SV40, JCV and BKV proteins, and drafted the manuscript; RD performed the stereotactic biopsy and made intellectual contributions to the manuscript; FBF was in charge of the inpatient management of the patient, and collected the data and analyzed it; RSN was in charge of the inpatient management of the patient, collected the data and analyzed it; EEC performed the morphological analysis and histopathological studies; CSP performed the PCR for BKV and JCV in CSF, and BKV sequence in CSF, and drafted the manuscript; JRT designed and drafted the manuscript, supervised the study, and made critical intellectual contributions; ACP was in charge of the inpatient/outpatient care, supervised the study, drafted the manuscript, analyzed the data, and made critical intellectual contributions.

## References

[B1] Trujillo JR, Jaramillo-Rangel G, Ortega-Martinez M, Penalva de Oliveira AC, Vidal JE, Bryant J, Gallo RC (2005). International NeuroAIDS: prospects of HIV-1 associated neurological complications. Cell Res.

[B2] Sacktor N (2002). The epidemiology of human immunodeficiency virus-associated neurological disease in the era of highly active antiretroviral therapy. J Neurovirol.

[B3] d'Arminio Monforte A, Cinque P, Mocroft A, Goebel FD, Antunes F, Katlama C, Justesen US, Vella S, Kirk O, Lundgren J (2004). Changing incidence of central nervous system diseases in the EuroSIDA cohort. Ann Neurol.

[B4] Oliveira JF, Greco DB, Oliveira GC, Christo PP, Guimaraes MD, Oliveira RC (2006). Neurological disease in HIV-infected patients in the era of highly active antiretroviral treatment: a Brazilian experience. Rev Soc Bras Med Trop.

[B5] Koralnik IJ, Wuthrich C, Dang X, Rottnek M, Gurtman A, Simpson D, Morgello S (2005). JC virus granule cell neuronopathy: A novel clinical syndrome distinct from progressive multifocal leukoencephalopathy. Ann Neurol.

[B6] Riedel DJ, Pardo CA, McArthur J, Nath A (2006). Therapy Insight: CNS manifestations of HIV-associated immune reconstitution inflammatory syndrome. Nat Clin Pract Neurol.

[B7] Vendrely A, Bienvenu B, Gasnault J, Thiebault JB, Salmon D, Gray F (2005). Fulminant inflammatory leukoencephalopathy associated with HAART-induced immune restoration in AIDS-related progressive multifocal leukoencephalopathy. Acta Neuropathol (Berl).

[B8] Gray F, Chretien F, Vallat-Decouvelaere AV, Scaravilli F (2003). The changing pattern of HIV neuropathology in the HAART era. J Neuropathol Exp Neurol.

[B9] Bratt G, Hammarin AL, Grandien M, Hedquist BG, Nennesmo I, Sundelin B, Seregard S (1999). BK virus as the cause of meningoencephalitis, retinitis and nephritis in a patient with AIDS. Aids.

[B10] Vallbracht A, Lohler J, Gossmann J, Gluck T, Petersen D, Gerth HJ, Gencic M, Dorries K (1993). Disseminated BK type polyomavirus infection in an AIDS patient associated with central nervous system disease. Am J Pathol.

[B11] Lesprit P, Chaline-Lehmann D, Authier FJ, Ponnelle T, Gray F, Levy Y (2001). BK virus encephalitis in a patient with AIDS and lymphoma. Aids.

[B12] Hirsch HH, Steiger J (2003). Polyomavirus BK. Lancet Infect Dis.

[B13] Arthur RR, Dagostin S, Shah KV (1989). Detection of BK virus and JC virus in urine and brain tissue by the polymerase chain reaction. J Clin Microbiol.

[B14] Behzad-Behbahani A, Klapper PE, Vallely PJ, Cleator GM (2003). BK virus DNA in CSF of immunocompetent and immunocompromised patients. Arch Dis Child.

[B15] Behzad-Behbahani A, Klapper PE, Vallely PJ, Cleator GM, Bonington A (2003). BKV-DNA and JCV-DNA in CSF of patients with suspected meningitis or encephalitis. Infection.

[B16] Hirsch HH (2005). BK virus: opportunity makes a pathogen. Clin Infect Dis.

[B17] Vago L, Cinque P, Sala E, Nebuloni M, Caldarelli R, Racca S, Ferrante P, Trabottoni G, Costanzi G (1996). JCV-DNA and BKV-DNA in the CNS tissue and CSF of AIDS patients and normal subjects. Study of 41 cases and review of the literature. J Acquir Immune Defic Syndr Hum Retrovirol.

[B18] McArthur JC, Haughey N, Gartner S, Conant K, Pardo C, Nath A, Sacktor N (2003). Human immunodeficiency virus-associated dementia: an evolving disease. J Neurovirol.

[B19] Cinque P, Bossolasco S, Brambilla AM, Boschini A, Mussini C, Pierotti C, Campi A, Casari S, Bertelli D, Mena M, Lazzarin A (2003). The effect of highly active antiretroviral therapy-induced immune reconstitution on development and outcome of progressive multifocal leukoencephalopathy: study of 43 cases with review of the literature. J Neurovirol.

